# A cluster randomized controlled trial for child and parent weight management: children and parents randomized to the intervention group have correlated changes in adiposity

**DOI:** 10.1186/s40608-017-0175-z

**Published:** 2017-12-04

**Authors:** Diane C. Berry, Robert G. McMurray, Todd A. Schwartz, Emily G. Hall, Madeline N. Neal, Reuben Adatorwover

**Affiliations:** 1 School of Nursing, The University of North Carolina at Chapel Hill, Campus Box 7460, Chapel Hill, NC 27599-7460 USA; 2 Department of Exercise and Sport Science, Department of Nutrition, The University of North Carolina at Chapel Hill, Campus Box 8700, Chapel Hill, NC 27599-8700 USA; 3 Department of Biostatistics, Gillings School of Global Public Health and School of Nursing, The University of North Carolina at Chapel Hill, Campus Box 7420, Chapel Hill, NC 27599-7420 USA

**Keywords:** Overweight, Obesity, Children, Parents, Rural, Low-income, Adiposity, Weight, Health behaviors, Self-efficacy

## Abstract

**Background:**

Studies have suggested that obesity is linked within families and that successful interventions involve both the parent and child with obesity. However little information exists regarding similarities in adiposity and weight loss between the parent and child, especially in low socio-economic ethnically diverse households.

**Methods:**

The purpose of this study was to examine the relationships between the changes from baseline over time in adiposity, weight, health behaviors, and self-efficacy in children (*n* = 184) and parents (*n* = 184) participating in an 18-month weight loss program. Within the intervention group only and for each post-baseline time point, Pearson correlation coefficients were computed for children’s changes (from baseline) in adiposity, weight, health behaviors, and self-efficacy, with their parents’ corresponding changes from baseline, to determine how strongly the dyads were correlated.

**Results:**

At the completion of 18 months, the intervention group parents demonstrated strong positive correlations between parent and child change in waist circumference (*r* = 0.409, *p* < 0.001), triceps (*r* = 0.332, *p* < 0.001), and subscapular (*r* = 0.292, *p* = 0.002) skinfolds. There were no significant correlations between weight, health behaviors, eating, and exercise self-efficacy.

**Conclusions:**

The results suggest that in the Southern United States low-income parents and their children with obesity are strongly correlated.

**Trial registration:**

NCT01378806 Retrospectively Registered on June 22, 2011.

## Background

Children and adults with overweight and obesity have increased dramatically over the past three decades [1]. African American and Hispanic children and adults are at especially high risk [1]. In the United States (U.S.), overweight or obesity affects 38% of children and 69% of adults in the general population [1]. Overweight and obesity are major contributors to premature morbidity and mortality due to type 2 diabetes and cardiovascular disease [2, 3]. Medical expenditures related to overweight and obesity now exceed $300 billion per year in the U.S. [4].

Treatments for children with overweight are designed to slow the rate of weight gain while achieving normal growth and development [5, 6]. Approaches found most effective have incorporated both behavioral and cognitive strategies [7] with parental involvement [8–12]. One study found that when parents learned eating behavior modification and had opportunities for exercise, their 6- to 11-year-old children showed a greater reduction in weight [13]. In another study, the involvement of at least one parent as an active partner in the weight loss process improved 8- to 12-year-old children’s short- and long-term weight regulation [14]. However, these interventions targeted middle-income, non-Hispanic white children. These reports have focused on the child, with minimal parental input and have been conducted primarily with non-Hispanic white, middle-income, 8- to12-year-old children in clinic settings [7].

We recently examined the effects of a two-phase intervention on adiposity, weight, health behaviors, and self-efficacy in second-to fourth-grade, low-income, rural, southern children in the U.S. matched with one of their parents. The primary and secondary outcomes for the study were published elsewhere [15]. For this manuscript, we examined the relationships between the changes from baseline in adiposity, weight, health behaviors, and self-efficacy in the group of children and parents randomized to the intervention group.

## Methods

### Design, settings, and sample

The study was a five-year, cluster randomized, controlled, community-based trial that partnered a second- to fourth-grade child with his or her parent with overweight or obesity and tested the efficacy of an innovative nutrition and exercise education, coping skills training, and exercise intervention. The University of North Carolina at Chapel Hill Institutional Review Board (# 07–0436) approved the study. A detailed description of the study design and recruitment has been published in the protocol manuscript [16]. The trial took place in the setting of eight elementary schools that were similar structure, size, and rural, ethnic, and racial mix. There were eight enrollment waves, with approximately 44–45 dyads of children and their parents enrolled in each wave, for a total of 358 children and 358 parents. All children had a body mass index (BMI) ≥ 85th percentile for age and gender; the ability to speak, write, and read in English; were seven to ten years of age and in the second to fourth grade; and at least one parent with a BMI ≥ 25 kg/m^2^. Parents provided written consent for themselves and their children and children provided written assent for themselves in the presence of their parents. Their parents could speak, write, and read in English and gave written and verbal consent to join the study. Dyads were excluded if comorbidities existed or they were participating in another weight management program [17].

### Intervention

The children and parents randomized to the intervention group received a two-phase intervention with follow-up, as described in the protocol publication [16]. Briefly, in Phase I (Intensive Intervention), the children and parents received 60 min of nutrition and exercise education and coping skills training and 45 min of exercise once a week for 12 weeks [5, 18, 19]. Exercise sessions included basketball, soccer, tag, walking, jumping rope, cardio kickboxing, Dance Revolution, strength training, and information on how to decrease sedentary behavior and increase exercise. Children and parents received a pedometer and a logbook, and they were encouraged to incrementally increase their activity to 10,000 steps per day. In Phase II (Continued Support), they met once each month for nine months with the interventionist to problem-solve issues they were having with nutrition and exercise. The intervention children and parents received 21 contacts over 12 months. They were then followed for six more months after the completion of Phase II, to assess the maintenance of results, for a total of 18 months in the study.

### Data collection

Data collection procedures have been described in-depth in the protocol publication [16]. Data were collected at four time points: Baseline was at enrollment; Post Phase I was 3 months post-baseline; Post Phase II was 12 months post-baseline; and the final data collection was after 6 months of no contact from the study staff or 18 months post-baseline.

A wait-listed control group of children and parents received usual care and had data collected at the same times as the intervention children and parents. After they completed the final data collection, they were offered the nutrition and exercise education, coping skills training, and exercise intervention (Phase I only). While this group was an important part of the trial design, this manuscript only focuses on the effects that occurred in those randomized to the intervention.

Data collected included demographics, adiposity, weight, health behaviors, and self-efficacy described elsewhere [16]. Height and weight were measured; BMI of parents and BMI percentile of children was calculated by computer using the Centers for Disease Control definitions and cutoffs [20]. The adiposity outcomes were waist circumference, triceps and subscapular skinfolds measured according to the National Health and Nutrition Examination Survey Anthropometry Procedures Manual [21].

To measure health behavior outcomes, the Adult Health Behavior Survey [22] and the Child Health Behavior Survey [22] were used to collect information on usual food and beverage intake, and the Child and Adolescent Trial for Cardiovascular Health (CATCH) questionnaire was used to measure diet and exercise behaviors in the children [23]. The Health Promoting Lifestyle Profile II was used to measure health behaviors in parents [24].

The Eating Self-Efficacy Scale [25] was used for the parents to measure negative affect (emotional eating) and socially acceptable circumstances (holidays) subscales. Exercise self-efficacy in parents was measured using Bandura’s Exercise Self-Efficacy Scale [26]. The CATCH questionnaire was used to measure eating and exercise self-efficacy in children [23].

### Data analysis

All analyses were performed using SAS, version 9.2 (SAS Institute Inc., Cary, NC). Within the intervention group only and for each post-baseline time point, Pearson correlation coefficients were computed for children’s mean changes (from baseline) in adiposity, weight, health behaviors, and self-efficacy, with their parents’ corresponding mean changes from baseline, to determine how strongly the dyads were correlated.

## Results

Cluster randomization resulted in 51% (*n* = 184) of the parent-child dyads assigned to the intervention group (Table 1). These parents’ mean age was 36.9 (SD +/−8.1) years. The majority of the parents were female, married, worked full-time, worked at technical jobs, and had a high school diploma or an Associate’s degree. The majority of parents were African Americans, earned less than $20,000 or between $20,000–39,999 per year, and were the biological parent of the child with whom they joined the study. These children’s mean age was 9.2 (SD +/− 0.96) years, and the majority were female, in third or fourth grade, and African American.

**Table 1:** Intervention group parents’ (*N* = 184) and their children’s (*N* = 184) baseline characteristics

Variable		Mean (Standard Deviation) or *N* and Percent	
Parent			
Age		36.9 (8.1)	
		N	Percent
Gender	Male	13	7.07
	Female	171	92.93
Marital Status			
	Married	86	46.74
	Widowed	2	1.09
	Divorced/Separate	29	15.76
	Never Married	50	27.17
	Living with Someone	17	9.24
Employment			
	Full-Time	101	54.89
	Part-Time	20	10.87
	Full-Time Student	10	5.43
	Homemaker	25	13.59
	Unemployed	25	13.59
	Retired	3	1.63
Occupation			
	Professional	39	21.20
	Technical	145	78.80
Education Level			
	6th Grade or Less	2	1.09
	Middle School	12	6.52
	High School/GED	65	35.33
	Associates	85	46.20
	Baccalaureate	14	7.61
	Graduate	6	3.25
Race			
	African American	117	63.59
	White	55	29.89
	Other	12	6.52
Income			
	< $20,000	65	35.33
	$20,000–$39,999	60	32.61
	$40,000–$59,999	22	11.96
	$60,000–$79,999	7	3.80
	$80,000–$99,999	7	3.80
	> = $100,000	1	0.54
	Do not wish to respond	22	11.96
Biological Parent			
	Yes	157	85.33
	No	27	14.67
Children			
Age		9.2 (+/− 0.96)	
		N	Percent
Gender	Male	83	45.10
	Female	101	54.90
Education Level at time of enrollment			
	2nd Grade	35	19.00
	3rd Grade	72	39.10
	4th Grade	77	41.90
Race			
	African American	117	63.60
	White	50	27.20
	Other	17	9.20

There was a significant correlation between parents’ and their children’s changes in waist circumference at Post Phase I (3 months; *r* = 0.328; *p* < 0.001), Post Phase II (12 months; *r* = 0.259; *p* = 0.005), and the completion of the study (18 months; r = 0.409; *p* < 0.001). See Table 2. In addition, there was also a significant correlation between parents’ and their children’s changes in triceps skinfolds at each of Post Phase I (*r* = 0.429; *p* < 0.001), Post Phase II (*r* = 0.533; *p* < 0.001) and at completion of the study (*r* = 0.332; *p* < 0.001) and subscapular skinfolds at Post Phase II (*r* = 0.368; *p* < 0.001) and at completion of the study (*r* = 0.292; *p* = 0.002). There was no significant correlation between parents’ and their children’s change in either weight or BMI at Time 2, Time 3, or Time 4. There was a significant correlation between parents’ and their children’s change in nutrition at Post Phase I (*r* = 0.203; *p* = 0.012), however, significance was not sustained at Post Phase II nor at completion of the study. There was no significant correlation between parents’ and their children’s changes in eating or exercise self-efficacy at any of the time points.

**Table 2:** Correlations between changes in intervention group children’s (*N* = 184) and parent’s (*N* = 184) outcomes from baseline at each study follow-up data collection

Variable	Post Phase I Intervention (3 Months)		Post Phase II Intervention (12 Months)		Completion of Study (18 Months)	
	r	P	r	P	r	P
Parent and Child Triceps Skinfolds (millimeter)	0.429	**<0.001**	0.533	**<0.001**	0.332	**<0.001**
Parent and Child Subscapular Skinfolds (millimeter)	0.151	0.065	0.368	**<0.001**	0.292	**0.002**
Parent and Child Waist Circumference (centimeter)	0.328	**<0.001**	0.259	**0.005**	0.409	**<0.001**
Parent and Child Weight (kilogram)	0.039	0.635	0.003	0.973	0.037	0.697
Parent and Child Weight (percent)	0.030	0.711	−0.008	0.930	0.053	0.575
Parent BMI and Child BMI Percentile	0.007	0.932	0.047	0.615	−0.114	0.235
Parent and Child Nutrition Knowledge and Behaviors	0.203	**0.012**	0.002	0.984	0.024	0.802
Parent and Child Eating Self-Efficacy	−0.067	0.411	−0.131	0.152	−0.111	0.239
Parent and Child Exercise Self-Efficacy	0.121	0.139	0.162	0.075	−0.098	0.297

r: Pearson Correlation Coefficient

p: *p*-value testing null hypothesis of correlation = 0; *p*-values significant at the 0.05 level are in **bold**

## Discussion

The results from this study are noteworthy because this is the first large study that examined the correlations of changes in adiposity, weight, health behaviors, and eating and exercise self-efficacy between a large cohort of low-income, rural children and their parents from the U.S. Previous studies have also noted the correlation of adiposity changes between children and parents [27–29]; however, these studies were not conducted with low-income, rural, majority African American children and parents. This study was notable in that changes in adiposity (waist circumference and triceps and subscapular skinfolds) for children and their parents were significantly correlated. Recent research has found that in children, adiposity has been found to be one of the first parameters to change in weight management interventions and may be a better measure of change when compared to BMI percentile change [30]. It is unclear why there were no significant correlations between children’s and parents’ changes in BMI. Management of overweight and obesity in children is focused on slowing the velocity of adiposity and weight gain and is designed to maintain normal growth and development [1]. In other studies, a strong correlation between BMI percentile in children and BMI in mothers has been found [28, 30]. The majority of the parents in this study were mothers. Mothers and their children were taught together in the same classroom, and the intervention was delivered at a second-grade literacy level. In contrast, our data suggests that BMI percentile may not be sufficiently sensitive to use in weight management studies in children, as previously noted [30]. This study adds to the literature suggesting that parents participating in a weight management program may also show similar changes in adiposity before significant changes in BMI. Thus, children and parents involved in a weight management program can decrease their adiposity regardless of BMI.

Equally interesting is why there were no significant positive correlations founds between changes in children’s and parents’ nutrition and exercise knowledge and behaviors, as they received the same information at the same time. One would expect that these young children received the majority of their knowledge and behaviors from their parents. However, there was no correlation between children’s and parents’ changes in nutrition and exercise knowledge and behaviors. This could be related to the relative importance assigned to the knowledge and behaviors by the parent as compared to children; the parents may have felt the information was more vital than the children. However, this is purely speculation based on anecdotal comments by the group.

It was not surprising that there were no significant correlations between children’s and parents’ changes in eating and exercise self-efficacy. Self-efficacy is one of the most difficult parameters to change, especially in children [26, 31–34]. The intervention was based on social cognitive theory [26, 31–34] and was designed to improve health knowledge and behaviors and increase self-efficacy in children and parents. It was believed that children and parents who developed skills in communication, goal setting, problem solving, conflict resolution and positive reinforcement would be more able to make healthy nutrition and exercise behavior change and manage their weight [26, 31–34]. However, it became clear that the parents and children due to school and work obligations were limited to choices throughout their days. In the main study [16], parents were interviewed at the completion of their time in the study and shared that their children arrived at school early, were fed a subsidized breakfast and lunch and were directed when they can go out to recess to play and for what duration. The majority of the children in this study went to after-school programs, where the pattern was repeated. They had little choice in what to eat and when they could be physically active. It was similar for their parents in that the majority of parents worked at low-income jobs when lunch breaks were predetermined and they had limited choices of healthy food and opportunities for exercise. They picked up their children from school and sometimes went through a fast-food drive-through because the prices were low, their children were hungry, and they were exhausted at the end of the day. Then they drove home, often to neighborhoods where it was unsafe for them or their children to go outside to exercise [16].

### Limitations

Limitations of the study include that the data do not reflect a representative sample of all overweight and obese children and parents. Data were self-reported, except for adiposity, and weight; therefore potential bias from under-reporting may have under-estimated the correlations may have occurred. Despite these limitations, the study offers notable strengths. This is a large sample of parent-child dyads providing important information on the correlations of changes in adiposity, weight, health behaviors, and self-efficacy in a group of overweight and obese low-income ethnically diverse children and parents in the rural southern U.S. The study also demonstrated the importance of combined child-parent weight loss interventions.

## Conclusions

In conclusion, our study highlights the importance of combined parent-child interventions and that it is possible to work with low-socioeconomic status children and parents who are already overweight or obese to improve adiposity in both. Future studies should focus on parents of younger children who have not yet become overweight or obese and work with the parents to decrease excessive adiposity and weight gain in their young children through healthy nutrition and exercise behaviors. The results further suggest that measures of adiposity, beyond BMI, need to be included in any weight management program or study. The study has implications for future community-based programs and include that adiposity is possibly a better measurement for children rather than BMI percentile in weight management interventions.

## References

[1] Centers for Disease Control and Prevention (2009). Estimated county-level prevalence of diabetes and obesity - United States, 2007. MMWR Morb Mortal Wkly Rep.

[2] American Diabetes Association (2017). American Diabetes Association standards of medical Care in Diabetes-2016. Diabetes Care.

[3] American Heart Association and American Stroke Association (2017). 2017 heart disease and stroke Statistics-2017 update: a report from the American Heart Association. Circulation.

[4] Behan DF, Cox SH, Lin Y, Pai J, Pedersen HW, Yi M. Obesity and its relation to mortality and morbidity costs. Society of Actuaries. 2010:1–61.

[5] U.S. Department Health and Human Services: Dietary Guidelines for Americans (2005). Chapter 3 weight management.

[6] National Center for Health Statistics. Health, United States, 2011: special feature on socioeconomic status and health. Edited by CDC. Hyattsville, MD. National Center for Health Statistics. 2012:1–583. 22812021

[7] Summerbell CD, Ashton V, Campbell KJ, Edmunds L, Kelly S, Waters E. Interventions for treating obesity in children: Cochrane review. Oxford: Wiley Publishers 2004;1–59. 10.1002/14651858.CD001872 12917914

[8] Bautista-Castano I, Doreste J, Serra-Majem L (2004). Effectiveness of interventions in the prevention of childhood obesity. Eur J Epidemiol.

[9] Wadden TA, Stunkard AJ, Rich L, Rubin CJ, Sweidel G, McKinney S (1990). Obesity in black adolescent girls: a controlled clinical trial of treatment by diet, behavior modification, and parental support. Pediatrics.

[10] Israel AC, Stolmaker L, Andrain CA (1985). The effects of training parents in general child management skills on a behavioral weight loss program for children. Behav Thera.

[11] Israel AC, Guile CA, Baker JE, Silverman WK (1994). An evaluation of enhanced self-regulation training in the treatment of childhood obesity. J Pediatr Psychol.

[12] Golan M, Fainaru M, Weizman A (1998). Role of behaviour modification in the treatment of childhood obesity with the parents as the exclusive agents of change. Int J Obes Relat Metab Disord.

[13] Golan M, Weizman A, Apter A, Fainaru M (1998). Parents as exclusive agents of change in the treatment of childhood obesity. Am J Clin Nutr.

[14] Epstein LH (1996). Family-based behavioural intervention for obese children. Int J Obes Rel Dis.

[15] Berry DC, Schwartz TA, McMurray RG, Skelly AH, Neal M, Hall EG, Aimyong N, Amatuli DJ, Melkus G (2014). The family partners for health study: a cluster randomized controlled trial for child and parent weight management. Nutr Diabetes.

[16] Berry DC, McMurray RG, Schwartz TA, Skelly AS, Sanchez M, Neal M, Hall EG (2012). Rationale, design, methodology and sample charactristics for the family Partners for Health Study: a cluster randomized controlled study. BMC Public Health.

[17] Nazare JA, Smith JD, Borel AL, Haffner SM, Balkau B, Ross R, Massien C, Alméras N, Després JP (2012). Ethnic influences on the relations between abdominal subcutaneous and visceral adiposity, liver fat, and cardiometabolic risk profile: the international study of prediction of intra-abdominal adiposity and its relationship with Cardiometabolic risk/intra-abdominal adiposity. Am J Clin Nutr.

[18] Forman SG (1993). Coping skills training for children and adolescents.

[19] American College of Sports Medicine. Guidelines for Exercise Testing and Prescription. 7th edition. New York: Lippincott Williams and Wilkins. 2006.

[20] What is BMI? http://www.bmi-calculator.net/.

[21] Heart N. Lung, and blood Istitute. The practical guide to identification, evaluation, and treatment of overweight and obesity in adults. Washington, DC: National Institutes of. Health. 2000:1–94.

[22] Department of Health and Human Services: Health Behavior Survey: Physical Activity and Nutrition (PAN) Behaviors Monitoring Form. Raleigh, North Carolina: Department of Health and Human Services. 2004.

[23] Parcel GS, Edmundson E, Perry CL, Feldman HA, O'Hara-Tompkins N, Nader PR, Johnson CC, Stone EJ. Measurement of self-efficacy for diet-related behaviors among elementary school children. J Sch Health 1995; 65(1):23–27. 10.1111/j.1746-1561.1995.tb03335.x 7731197

[24] Walker SN, Sechrist KR, Pender NJ (1987). The health-promoting lifestyle profile: development and psychometric characteristics. Nurs Res.

[25] Glynn SM, Ruderman J (1986). The development and validation of an eating self-efficacy scale. Cognit Ther Res.

[26] Bandura A. Self-efficacy. The exercise of control. New York: WH. Freeman. 1997;

[27] Summerbell CD, Waters E, Edmunds L, Kelly S, Brown T, Campbell KJ. Interventions for preventing obesity in children. Cochrane Reviews. Cochrane Database of Systematic Reviews 2005(3):Art. No.: CD001871. doi:10.001002/14651858.CD14001871.pub14651852. 10.1002/14651858.CD001871.pub2 16034868

[28] Oude Luttikhuis H, Baur L, Jansen H, Shrewsbury VA, O'Malley C, Stolk RP, Summerbell CD. Interventions for treating obesity in children. Cochrane Database of Systematic Reviews 2009 2009(1):Art. No.: CD001872. doi:10.001002/14651858.CD14001872.pub14651852. 10.1002/14651858.CD001872.pub2 19160202

[29] Power C, Pouliou T, Li L, Cooper R, Hyppönen E (2011). Parental and offspring adiposity associations: insights from the 1958 British birth cohort. Ann Hum Biol.

[30] Kolotourou M, Radley D, Chadwick P, Smith L, Orfanos S, Kapetanakis V, Singhal A, Cole TJ, Sacher PM, BMI I (2013). Alone a sufficient outcome to evaluate interventions for child obesity?. Child Obes.

[31] Bandura A (1977). Social learning theory.

[32] Bandura A (1986). Social foundations of thought and action: a social cognitive theory.

[33] Bandura A (1982). Self-efficacy mechanism is human agency. Am Psychol.

[34] Bandura A (2004). Health promotion by social cognitive means. Health Educ Behav.

