# Correction to: A cluster randomized controlled trial for child and parent weight management: children and parents randomized to the intervention group have correlated changes in adiposity

**DOI:** 10.1186/s40608-018-0181-9

**Published:** 2018-01-26

**Authors:** Diane C. Berry, Robert G. McMurray, Todd A. Schwartz, Emily G. Hall, Madeline N. Neal, Reuben Adatorwovor

**Affiliations:** 1 School of Nursing, The University of North Carolina at Chapel Hill, Campus Box 7460, Chapel Hill, NC 27599-7460 USA; 2 Department of Exercise and Sport Science, Department of Nutrition, The University of North Carolina at Chapel Hill, Campus Box 8700, Chapel Hill, NC 27599-8700 USA; 3 Department of Biostatistics, Gillings School of Global Public Health and School of Nursing, The University of North Carolina at Chapel Hill, Campus Box 7420, Chapel Hill, NC 27599-7420 USA

## Erratum

After publication of the original article [1] it was highlighted that the surname of author Reuben Adatorwovor was incorrectly typeset as Adatorwover. These errors were introduced during typesetting; thus the publisher apologizes for this error. Additionally, the original manuscript has also been updated to amend this error.
